# Evaluation of the Reliability, Reproducibility and Validity of Digital Orthodontic Measurements Based on Various Digital Models among Young Patients

**DOI:** 10.3390/jcm9092728

**Published:** 2020-08-24

**Authors:** Seo-Hyun Park, Soo-Hwan Byun, So-Hee Oh, Hye-Lim Lee, Ju-Won Kim, Byoung-Eun Yang, In-Young Park

**Affiliations:** 1 Division of Pediatric Dentistry, Hallym University Sacred Heart Hospital, Anyang 14066, Korea; park070676@gmail.com (S.-H.P.); colfman@hanmail.net (S.-H.O.); onlylove0210@naver.com (H.-L.L.); 2 Graduate School of Clinical Dentistry, Hallym University, Chuncheon 24252, Korea; purheit@hallym.or.kr (S.-H.B.); kjw9199@hallym.or.kr (J.-W.K.); 3 Institute of Clinical Dentistry, Hallym University, Chuncheon 24252, Korea; 4 Division of Oral and Maxillofacial Surgery, Hallym University Sacred Heart Hospital, Anyang 14066, Korea; 5 Division of Orthodontics, Hallym University Sacred Heart Hospital, Anyang 14066, Korea

**Keywords:** digital model, intraoral scanner, intraoral scanned digital model, digital orthodontic measurement, tooth width, arch length, arch length discrepancy

## Abstract

The advantages of intraoral model scanning have yielded recent developments. However, few studies have explored the orthodontic clinical use of this technique particularly among young patients. This study aimed to evaluate the reliability, reproducibility and validity of the orthodontic measurements: tooth width, arch length and arch length discrepancy in each digital model obtained by model scanner and intraoral scanner, relative to a plaster model. Arch length measured using two methods: curved arch length (CAL) measured automatically by digital program and sum of sectional liner arch length (SLAL) measured sum of anterior and posterior liner arch lengths. Arch length discrepancy calculated each arch length measurement methods: curved arch length discrepancy (CALD) and sum of sectional liner arch length discrepancy (SLALD). Forty young patients were eligible for the study. A plaster model (P), model-scanned digital model (MSD) and intraoral scanned digital model (ISD) were acquired from each patient. The reliability of the measurements was evaluated using Pearson’s correlation coefficient, while the reproducibility was evaluated using the intraclass correlation coefficient. The validity was assessed by a paired t-test. All measurements measured in P, MSD and ISD exhibited good reliability and reproducibility. Most orthodontic measurements despite of CAL in MSD exhibited high validity. Only the SLAL and SLALD in ISD group differed significantly, despite the good validity of the tooth width, CAL and CALD. The measurements based on the digital program appeared high reliability, reproducibility and accurate than conventional measurement. However, SLAL and SLALD in ISD group appeared shorter because of distortion during intraoral scanning. However, this could be compensated by using digital programed curved arch. Although the validity of SLAL and SLALD in the ISD group differed statistically, the difference is not considered clinically significant. Although MSD and ISD are acceptable for a clinical space analysis, clinicians should be aware of digital model-induced errors.

## 1. Introduction

Accurate measurements and a study model analysis are crucial components of a successful orthodontic treatment. Although treatment planning requires an analysis of crowding and spacing in the patient’s mouth, measurements of the tooth width (TW) and arch length (AL) are highly recommended as determinants of the arch length discrepancy (ALD) [1]. A plaster model is typically used for standard studies. However, this type of model has some disadvantages related to volumetric deformation, which can lead to errors, and is more likely to be damaged during storage and transportation [2,3]. Comparatively, a digital model has some advantages such as the ease of production and storage, good mobility and long-term economic benefits. In addition, a digital model provides immediate access to 3D data [4].

Three main methods are used to obtain the digital models: scanning of a plaster model with a 3D model scanner, direct scanning of the oral cavity with an intraoral scanner and obtaining a model via cone-beam computed tomography (CBCT). Previous studies demonstrated that a model-scanned digital model (MSD) produced using a 3D model scanner shows high accuracy with the conventional plaster model [4,5,6,7]. Additionally, the clinical compatibility and validity of MSD were approved for orthodontic measurements such as the size of the teeth, length and the width of the arch, Bolton ratio and occlusion [5,6,8,9,10]. Some researchers reported that the MSD provided a better accurate depiction than a plaster model [11]. MSDs have several advantages, but also share some disadvantages with plaster models, including undercutting and distortion of the impression around the bracket, as well as induction of the gagging reflex. However, intraoral scanners can resolve these disadvantages to some extent, which has led to rapid development in the field of intraoral scanned digital models (ISD). Specifically due to these advantages, many dental college hospitals are increasingly using various types of intraoral scanners for a variety of patients and are providing education and research to numerous dentists and medical personnel.

Some previous studies reported that ISD provides a higher level of accuracy with the MSD or plaster model at shorter scan lengths. However, the accuracy of the whole arch model was relatively poor when compared to plaster models and it remains uncertain whether such models could be used in clinical prosthodontics applications [12,13,14]. In addition, in the field of oral and maxillofacial surgery, research is being conducted on the use of models acquired with intraoral scanners in various orthognathic and implant surgery [15,16]. Further, earlier studies observed that the clinical use of the full arch should be approached carefully because of the risk of distortion when scanning more than half of the arch length [14,17,18,19,20,21].

Although existing orthodontics studies have explored the clinical validity of ISDs, only a few studies have comprehensively evaluated the reliability and reproducibility with validity of these models [22,23,24,25]. No studies have yet analyzed and compared orthodontic measurement aspects, such as arch length discrepancy, through the full arch by MSD and ISD among younger patients. This study aimed to evaluate the reliability, reproducibility and validity of the TW, AL and ALD as measured using different two methods, the sum of anterior and posterior sectional liner arch and automatically designed curved arch, on an MSD and ISD relative to a plaster model to determine the clinical application for a space analysis.

## 2. Materials and Methods

### 2.1. Patients Inclusion Criteria and Group Classification

**Figure 1:**
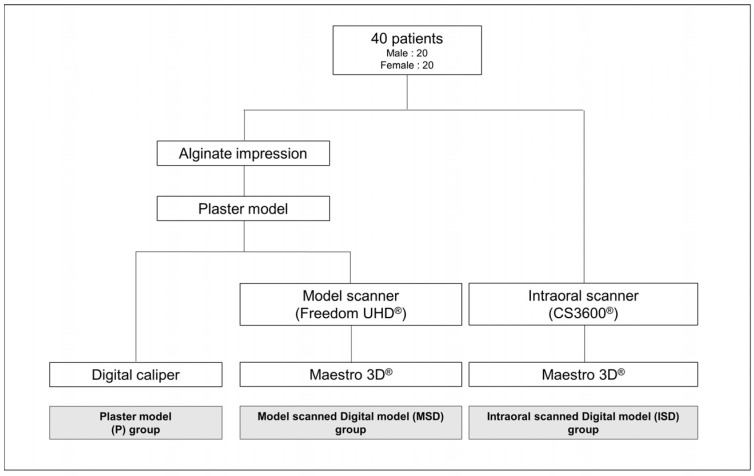
Illustration of the flow chart of model production according to model type.

The study population comprised 40 patients who visited the Hallym Sacred Heart Hospital Dental Clinic for an orthodontic diagnosis during January 2018–January 2019. The collection of the patients’ data was approved by the Institutional Review Board (IRB) of Hallym University Medical Center (IRB number: 2019-08-006-001). The number of specimens required for this study was estimated using a significance level of α = 0.05, 95% power and an effect size of 0.80 at G power (version 3.010, Franx Faul. Universitat Kiel, Germany). The sample included 20 male and 20 female patients aged 12–18 years; the mean ages of males and females were 13.6 and 12.7 years, respectively. The inclusion criteria were full eruption to the first permanent molar, no history of restoration or orthodontic treatment with no maxillofacial deformity and no missing or malformed teeth. Figure 1 depicts the process of model development for each patient.

**Figure 2:**
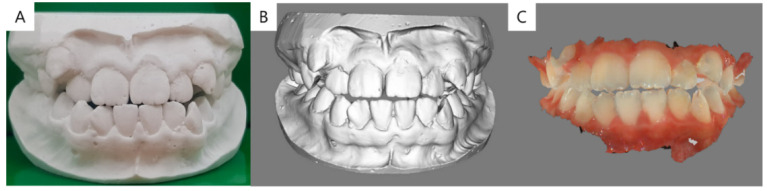
A representative plaster model and images scanned using a model scanner and intraoral scanner. (**A**) Plaster model. (**B**) Stereolithography language (STL) file format image scanned using the Freedom UHD^®^ 3D scanner(Dof Inc., Seongdong-gu, Seoul, Korea) (**C**) Object code (OBJ) file format image scanned using CS3600^®^(Carestream Dental, Atlanta, GA, USA). The latter was converted to a STL file for measurements.

First, a plaster model was obtained from each patient using an impression taken with alginate (Cavex Impressional; Cavex Holland BV, Haarlem, The Netherlands) and was immediately produced by white stone (Ryoka Dental; Mie-Ken, Japan) in proportions according to the manufacturer’s instructions (Figure 2A). Examiner A, who had sufficient education and experience in scanning, produced a MSD using a Freedom UHD^®^ 3D scanner (Dof Inc., Seongdong-gu, Seoul, Korea) according to the manufacturer’s protocol same condition on every model. An experienced dentist with sufficient scanning experience then scanned the patient’s ISD and obtained measurements using a CS3600^®^ intraoral scanner and software (Carestream Dental, Atlanta, GA, USA). As indicated by the manufacturer, scanning was initiated at the anterior labial surface to the lingual surface area and followed the buccal and occlusal surfaces of the posterior teeth to the left and right lingual surfaces. All digital models were saved as stereolithography language (STL) files, which are approved by the indicated software (Figure 2B,C).

### 2.2. Orthodontic Measurements

Measurements were performed by 4 specialists of orthodontics of Hallym Sacred Heart Hospital dental clinic who were sufficiently trained in the indicated methods. Examiner A performed 2 sets of measurements at an interval of 2 weeks. Vernier calipers (CD-20PSX, Mitutoyo Corp, Kawasaki, Japan) with a self-tolerance error of <0.02 mm were used to measure the plaster model. Maestro 3D dental studio^®^ (AGE Solutions, Pisa, Italy) software was used for digital model measurements. All measurements were made in 0.01 mm increments.

**Figure 3:**
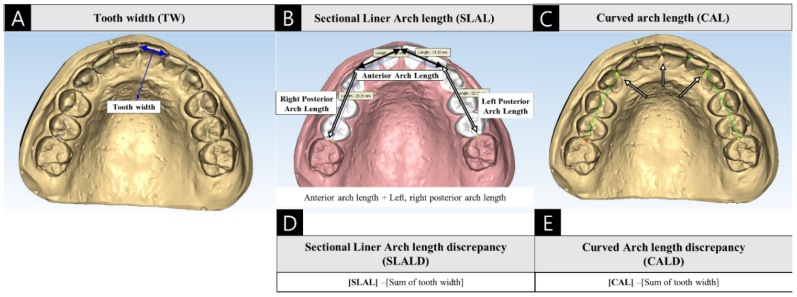
(**A**) Measurement of the mesial-distal width of the tooth and the arch length. (**B**) Sectional liner arch length (SLAL) measured using Maestro 3D^®^. (**C**) Automatically designed curved arch length (CAL) using Maestro 3D^®^. (**D**). Definition of sectional full arch length discrepancy (SLALD). (**E**) Definition of digital full arch length discrepancy (CALD).

The following model analysis criteria were applied. The AL and ALD were analyzed for each arch. For all teeth, the TW was measured as the longest mesial-distal width parallel to the occlusal plane (Figure 3A). In the digital model, the measurement point was set at the height of the contour, and recalibration was practiced using a coronal 2-dimensional sectional view. The AL was measured in 2 ways. First, the sectional liner arch length (SLAL) was defined as the sum of the anterior arch length (AAL), left arch length (LAL) and right arch length (RAL) and was measured parallel to the occlusal plane. The AAL was defined as the linear distance between the midpoint of the mesial height of the contour of both central incisors to the mesial height of the contour of each canine, and the LAL and RAL were defined as the linear distances between the mesial height of the contour of the canine to the mesial height of the contour of the first permanent molar on the indicated side (Figure 3B). Second, the digital curved arch length (CAL) was defined as the total length of the arch formed from the left mesial surface of the first permanent molar to the corresponding point on the other side of the automatically constructed arch curve (Figure 3C). This was determined using the mesial surface of each first permanent molar and canine and the center point of the central incisor and the digital arch length measuring mode in the Maestro 3D dental studio^®^ (AGE Solutions, Pisa, Italy) program.

The ALD was defined as the difference between the available space (AS) and required space (RS). The AS was defined as the AL of each arch, and the RS was defined as the sum of the tooth mesial-distal width from the left second premolar to the right second molar for each arch. Each SLAL and CAL were calculated together with the sectional full arch length discrepancy (SLALD) and digital full arch length discrepancy (CALD) (Figure 3D,E).

### 2.3. Statistical Methods

The collected data were subjected to statistical processing using the IBM SPSS Statistics software program (Version 24.0, IBM SPSS Inc., Chicago, IL, USA). The reliability was analyzed using Pearson’s correlation coefficients of the measured TW, AAL, LAL, RAL and CAL. The significance level of Pearson’s correlation coefficients was verified as *p* < 0.0001. Subsequently, the reproducibility was evaluated using the intraclass correlation coefficients (ICC) between the data collected by Examiners A, B, C and D. An ICC ≥ 0.9 was considered to indicate excellent reproducibility, while values of 0.75 to <0.9, 0.5 to <0.75 and <0.5 were considered good, moderate and poor, respectively. Finally, the validity of the space analysis, including the SLAL, CAL and CALD, was evaluated using a paired t-test to explore the differences between the Group P and Groups MSD and ISD. The first value measured by examiner A was reported as the representative value if the reliability and reproducibility were good.

## 3. Results

### 3.1. Reliability

Nearly all data collected by examiner A were highly reliable (Table 1, *p* < 0.0001). Very high reliability was also observed for the AAL, LAL and RAL in all 3 groups as measured by examiner A (*p* < 0.0001). For the mandible AAL, the highest reliability was observed in Group MSD. For the CAL, good reliability was achieved in all 3 groups (Table S1).

### 3.2. Reproducibility

All data from Examiner A, B, C and D exhibited significant reproducibility (Table S2). For the TW, the values measured by examiner A, B, C and D exhibited excellent or good reproducibility in all 3 groups. Although the ICCs in all groups indicate adequate reproducibility, Groups MSD and ISD yielded higher levels of reproducibility than Group P. The AAL, LAL, RAL and CAL measured by examiners A, B, C and D also exhibited significant reproducibility in all 3 groups. For AAL, LAL and RAL, the ICCs at the anterior and posterior teeth were excellent or good in Group P, good in Group MSD and moderate or good in Group ISD. For the CAL, however, the ICCs determined for Groups MSD and ISD were excellent.

### 3.3. Validity

In Group MSD, every measured TW was longer than the corresponding value in Group P, except for the lower left canine and first premolar. The greatest error was measured in the upper right first premolar (Table 1). The average error ranged from −0.123 to 0.001 mm, and the standard variation ranged 0.196 from 0.278. Both upper central incisors, the upper right lateral incisor and canine, both lower lateral incisors, the lower left canine and the first premolar seemed to be measured similarly.

In Group ISD, every measured TW was longer than the corresponding value in Group P, except the upper right central incisor and canine, upper left lateral incisor, lower left canine and first premolar. The average of error ranged from −0.106 to 0.078 mm, and the standard variation ranged from 0.196 to 0.278. Both upper central incisors and lateral incisors, the lower central incisor, lateral incisor, canine and first premolar and lower left first premolar seemed to be measured similarly. The tooth with the greatest error was upper left first premolar.

Regarding the SLAL between Groups P and MSD, the average error values were similar with a maxilla SLAL of 0.013 mm and a mandible SLAL of 0.060 mm. Neither difference was significant. However, the data from the maxilla and mandible LAL and RAL yielded significantly greater average error values ranging from −0.209 to −0.136 (Table 1, *p* < 0.05). However, the SLAL values of −0.399 mm in the maxilla and −0.236 mm in the mandible were not significant (Table 2).

Regarding the SLAL between Groups P and ISD, the average error values were significantly higher in the latter relative to the former, with a maxilla SLAL of 0.389 mm and mandible SLAL of 0.270 mm. However, the maxilla and mandible LAL and RAL measurements were similar between the groups, with no significant differences (average error: −0.136 to 0.133 mm; Table 1). The SLAL error values of 0.519 mm in the maxilla and 0.523 mm in the mandible indicated that this value was significantly lower in Group ISD than in Group P (Table 2, *p* < 0.05).

### 3.4. Validity of the Space Analysis

When the SLALD was measured, the average error values between Groups P group and MSD were 0.250 mm in the maxilla and 0.221 mm in the mandible, and these differences were not significant (Table 2). However, the CAL was significantly higher in Group MSD because of the differences resulting from curved and straight measurements. The average error value between Groups P and ISD were 0.752 mm in the maxilla and 0.676 mm in the mandible, and both SLALD values were significantly smaller in the ISD group than in the P group (Table 2, *p* < 0.05).

When the CALD was measured, the average error values between Groups P and MSD were0.106 mm in the maxilla and 0.193 mm in the mandible, and these differences were not significant (Table 2). The average error values between Groups P and ISD were 0.255 mm in the maxilla and0.040 mm in the mandible, and neither difference was significant (Table 2, *p* < 0.05).

## 4. Discussion

This study evaluated the reliability, reproducibility, and validity of the TW and AL measured using the MSD and ISD and the analyzed ALD in 40 young patients with permanent dentition. Notably, the TW, AAL, LAL and RAL measured in Groups P, MSD and ISD were very highly reliable and adequately reproducible. In the validity evaluation, most TW, AL and ALD values in Group MSD group and the TW values in Group ISD were highly valid. In comparison, the AL and ALD in Group ISD had relatively low validity, and the average total AL values in the maxilla and mandible were significantly smaller than those in Group P by 0.519 and 0.523 mm, respectively. Similarly, the average ALD values in the maxilla and mandible in Group ISD were significantly smaller than those in Group P by 0.752 and 0.676 mm, respectively.

An intraoral scanner can more efficiently evaluate oral conditions in patients with an undercut, orthodontic appliance, gag reflex or thicker soft tissue, which can increase the difficulty and reduce the accuracy of impression modeling [7,20,26]. Particularly, conventional impression modeling induces anxiety and the gagging reflex in pediatric patients, who find oral scanner-based modeling to be relatively more comfortable [17,20,27,28]. The proportion of pediatric orthodontic patients who begin orthodontic treatment at an early age has increased recently in response to the emerging aesthetic emphasis on an ideal appearance and dentition, and the use of an intraoral scanner, rather than an impression, could reduce discomfort and improve the process of orthodontic diagnosis and treatment [26,28]. An ISD is digitalized and saved immediately, which could also reduce the time required for the digital setup of a clear aligner and other orthodontic appliances and decrease the error associated with the impression step [23,29]. In the future, it would be preferable for additional research to confirm the reproducibility of the intra-premises scan model performed by individuals of various occupations (i.e., dental hygienists, nurses, students), rather than dentist-guaranteed.

Measurements of lengths in a diagnostic model are influenced by factors such as the contact between adjacent teeth, the undercut of the teeth, intraoral orthodontic appliances or soft tissue. Regarding contact methods, Vernier caliper measurements of plaster models provide better accuracy. However, a non-contact measurement method is recommended when the measurement range is wide or the measurement location is easily deformed because the accuracy of a contact method decreases when the measurement area is complex or difficult to reach. The digital model measurement is a non-contact method and can accurately reach areas that are difficult to access using a Vernier caliper or that are affected by severe crowding or orthodontic appliances. Digital models also enable a visual determination of the 3D tooth inclination or axis [30]. The height of the arch height, difference in height between marginal ridges, angles between teeth, overjet and overbite tend to be measured inaccurately by Vernier calipers. In such cases, 3D methods can provide more accurate measurements. Although digital measurement methods may require significant learning curves and adjustment periods, these methods have the advantage of saving considerable time for both patients and clinicians [17,20]. In addition, the point where the measurement was performed can be recorded, which may be helpful for future re-measurements.

In this study, the use of the Maestro 3D dental studio^®^ (AGE Solutions, Pisa, Italy) provided higher levels of reliability and reproducibility in terms of the TW measurements when compared to the Vernier caliper-based measurements. Specifically, the digital method was highly reproducible. Potentially, the coronal sectional view with a 3D tooth axis, which can be automatically visualized during measurement via a digital measurement program, could enable a set measurement point and help the clinician to measure this point more reproducibly, compared to a plaster model. Further, future research should confirm digital measurement while analyzing various digital programs. The superimposition of a digital model obtained from CBCT on this digital model, could further enable the more accurate measurement of the position of the tooth and the angle of the root in the alveolar bone.

The digital scanners used currently in clinical settings form the measurements using laser reflections. However, the laser may be reflected diffusely in areas such as adjacent surfaces, which can increase the possibility of error and reduce the reproducibility [31,32]. When the model scanner was used, the plaster model was constantly irradiated with lasers in various directions, and additional areas were scanned to increase the accuracy. However, a plaster model can easily cause physical errors due to air bubbles or stone surface defects, and impressions may be deformed or distorted at the undercut due to structural dental issues such as orthodontic appliances and interdental areas.

The digital model cannot be set as an accurate measurement point if it cannot directly reach the surface. Therefore, the ability to measure areas without data (e.g., inside bubbles) is limited. In this study, we observed significant differences in the LAL and RAL in Group MSD. These values were presumed to have been affected by the validity of the measurement points on adjacent surfaces, which were due to the error in the impression of the adjacent surfaces. In Group MSD, the SLAL tended to be measured overall, but again, the values did not differ significantly from those in Group P. Presumably, the error between the measurement points resulting from those measured in parts was reduced, which eliminated any significant differences.

**Figure 4:**
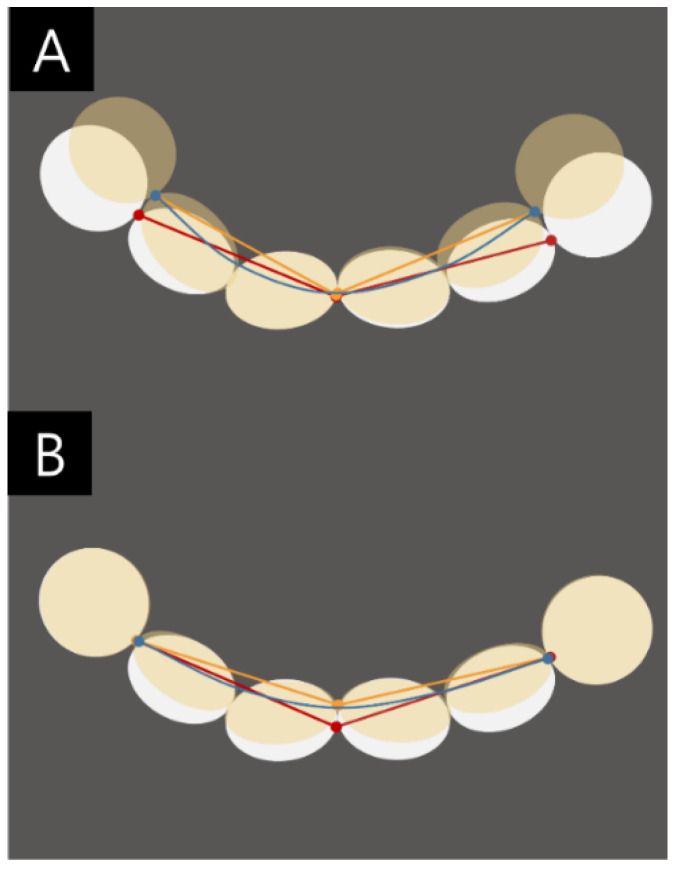
Illustration of differences in the sectional anterior arch length measurement caused by a distortion of intraoral scanning (**A**) and arch shape distortion. (**B**). Tooth labial-lingual width distortion. Red line: actual, yellow line: difference by distortion, blue line: curved arch.

In comparison, the ISD can provide digital images without defects from brackets or adjacent surfaces, which often cause surface defects or bubbles in stone models. Here, the TW measurements in Group ISD were most valid, indicating that the accurate measurement of a short span (e.g., TW) affected the impression without damaging the proximal and tooth surfaces. However, the SLAL value was significantly smaller in Group ISD than in Group P. Particularly, a significant error occurred in the AAL in Group ISD, and this may be due to the scanning method recommended by the CS3600^®^ (Carestream Dental, Atlanta, GA, USA) manufacturer. Further, when digitalizing the anterior dental arch during the intraoral scanning process, the shape and size of the straight head of the intraoral scanner prevents a smooth transition from the labial surface to the lingual surface. Another cause of errors in intraoral scans is distortion that occurs during the process of superimposition of the scanned images. As a result, it is a known issue that errors appear larger in a long span scan image [12,18,22,33,34]. Although the tooth shape can be obtained relatively accurately, the errors are thought to arise in the form or length of the arch due to distortions in the labial width, alveolar bone shape and occlusal plane of the arch form. We expect that this distortion was caused by the intraoral scan rather than by a digital measurement issue (e.g., an error in the approach to the measuring point) (Figure 4). However, we attributed the similarity to the ability to measure the length of the curved form, which was difficult to measure in the stone model. In other words, the error of the straight-line distance measured by the intraoral scanner can be compensated by measuring the curved distance.

In addition to the scanning method, errors may be caused by saliva, surrounding soft tissues, patient coordination and movement, the scanner head size, acquisition time, acquisition skill and acquisition range. Accordingly, further studies of the clinical validity of the ISD are warranted. Our study evaluated the validity of the measured values corresponding to the full arch span.

In last years, some studies evaluated clinical reliability and validity of the 3D measurements of features such as the palatal volume, occlusal relation, arch height, overjet and overbite, which are difficult to measure accurately using a Vernier caliper and stone models in MSD or CBCT digital model [35]. A study of the validity of 3D measurements in ISD would also be helpful.

Although we observed significant errors in the SLAL and SLALD in Group ISD, the relatively small error values in the maxilla and mandible would not significantly affect a diagnostic and treatment plan. However, the CAL and CALD produced using the digital program were deemed highly valid for clinical use. Therefore, dental analytic measurements based on an ISD may be clinically acceptable. The MSD is recommended for an orthodontic diagnosis that requires a measurement over a long span (e.g., full arch or alveolar ridge width) and a digital model for storage. The ISD is recommended when the predicted error of a stone model would be large due to the difficulty of impression taking, the interdental area or the presence of an orthodontic appliance. Moreover, if a space analysis such as the ALD is required in ISD, a digital program could be used to analyze the CAL and CALD while reducing the error. The resulting data would be highly valid for an orthodontic diagnosis.

## 5. Conclusions

In summary, the TW values obtained in Group ISD were more valid than those obtained in Group MSD. However, the former group yielded less valid SLAL and SLALD values. The CAL and CALD values in both groups exhibited high validity when compared with the corresponding values in Group P. Therefore, clinicians should be aware of the errors that may occur when using an MSD or ISD for a spatial analysis during the course of diagnosis and treatment. The appropriate impression method should be determined according to the individual situation.

## Figures and Tables

**Table 1:** Validity of tooth widths and arch lengths measured using each digital model.

Verification	p-MSD			P-ISD			Verification	P-MSD			P-ISD		
	Mean	SD	*p* Value	Mean	SD	*p* Value		Mean	SD	*p* Value	Mean	SD	*p* Value
#15	−0.088	0.223	0.000 ***	−0.096	0.297	0.001 **	#35	−0.061	0.251	0.009 **	−0.017	0.312	0.542
#14	−0.123	0.250	0.000 ***	−0.105	0.296	0.000 ***	#34	0.001	0.218	0.947	0.020	0.264	0.418
#13	−0.075	0.222	0.000 ***	0.078	0.293	0.004 **	#33	0.001	0.265	0.959	0.035	0.319	0.227
#12	−0.042	0.216	0.037 *	−0.007	0.231	0.747	#32	−0.034	0.196	0.056	−0.033	0.197	0.070
#11	−0.023	0.197	0.207	0.028	0.317	0.340	#31	−0.061	0.200	0.001 ***	−0.008	0.268	0.731
#21	−0.033	0.226	0.116	−0.011	0.254	0.358	#41	−0.063	0.202	0.001 ***	−0.026	0.222	0.201
#22	−0.034	0.258	0.150	0.002	0.282	0.954	#42	−0.039	0.242	0.082	−0.008	0.264	0.751
#23	−0.040	0.239	0.068	−0.056	0.270	0.024 *	#43	−0.070	0.278	0.007 **	−0.032	0.342	0.310
#24	−0.107	0.226	0.000 ***	−0.106	0.251	0.000 ***	#44	−0.062	0.225	0.003 **	−0.056	0.280	0.032 *
#25	−0.086	0.255	0.000 ***	0.060	0.271	0.008 **	#45	−0.070	0.260	0.004 **	−0.029	0.275	0.259
Maxilla							Mandible						
AAL	0.013	1.244	0.908	0.389	1.497	0.005 **	AAL	0.060	1.055	0.535	0.270	1.046	0.006 **
LAL	−0.204	0.882	0.013 *	0.076	1.094	0.451	LAL	−0.136	0.832	0.015 *	0.133	1.178	0.219
RAL	−0.209	1.119	0.043 *	0.045	1.085	0.146	RAL	−0.160	0.730	0.018 *	0.120	0.844	0.121

Paired *t*-test (*: *p* < 0.05, **: *p* < 0.01, ***: *p* < 0.001, unit = mm). P = plaster, MSD = model scanned digital model, ISD = intraoral scanned digital model, AAL = anterior arch length, LAL = left arch length, RAL = right arch length, CAL = curved arch length, SD = standard deviation.

**Table 2:** Validity of arch length and arch length discrepancy measured by two methods.

	P-MSD			P-ISD				P-MSD			P-ISD		
	Mean	SD	*p* Value	Mean	SD	*p* Value		Mean	SD	*p* Value	Mean	SD	*p* Value
Maxilla							Mandible						
SLAL	−0.399	2.328	0.063	0.519	2.695	0.000 *	SLAL	−0.236	1.641	0.118	0.523	2.070	0.007 *
CAL	−0.755	2.113	0.000 *	0.022	1.695	0.059	CAL	−0.649	2.013	0.000 *	−0.173	1.887	0.067
RS	−0.649	1.120	0.000 *	−0.233	1.234	0.197	RS	−0.456	1.191	0.000 *	−0.153	1.351	0.216
SLALD	0.250	2.410	0.257	0.752	3.084	0.009 *	SLALD	0.221	1.789	0.179	0.676	2.390	0.002 *
CALD	−0.106	2.998	0.321	0.255	2.929	0.117	CALD	0.193	2.410	0.257	−0.040	1.084	0.718

Paired *t*-test (*: *p* < 0.05, unit = mm), RS = required space, SLAL = sectional liner arch length, CAL = curved arch length, SLALD = sectional liner arch length discrepancy, CALD = curved arch length discrepancy.

## Supplementary Materials

The following are available online at https://www.mdpi.com/2077-0383/9/9/2728/s1, Table S1: Reliability of tooth widths and arch lengths in each group; Table S2: Reproducibility of the tooth widths and arch lengths in each group.

## References

[1] Richter A.E., Arruda A.O., Peters M.C., Sohn W. (2011). Incidence of caries lesions among patients treated with comprehensive orthodontics. Am. J. Orthod. Dentofac. Orthop..

[2] Crosby D.R., Alexander C.G. (1989). The occurrence of tooth size discrepancies among different malocclusion groups. Am. J. Orthod. Dentofac. Orthop..

[3] Schirmer U.R., Wiltshire W.A. (1997). Manual and computer-aided space analysis: A comparative study. Am. J. Orthod. Dentofac. Orthop..

[4] Motohashi N., Kuroda T. (1999). A 3D computer-aided design system applied to diagnosis and treatment planning in orthodontics and orthognathic surgery. Eur. J. Orthod..

[5] Zilberman O., Huggare J.A., Parikakis K.A. (2003). Evaluation of the validity of tooth size and arch width measurements using conventional and three-dimensional virtual orthodontic models. Angle Orthod..

[6] Fleming P.S., Marinho V., Johal A. (2011). Orthodontic measurements on digital study models compared with plaster models: A systematic review. Orthod. Craniofac. Res..

[7] Rossini G., Parrini S., Castroflorio T., Deregibus A., Debernardi C.L. (2016). Diagnostic accuracy and measurement sensitivity of digital models for orthodontic purposes: A systematic review. Am. J. Orthod. Dentofac. Orthop..

[8] Santoro M., Galkin S., Teredesai M., Nicolay O.F., Cangialosi T.J. (2003). Comparison of measurements made on digital and plaster models. Am. J. Orthod. Dentofac. Orthop..

[9] Stevens D.R., Flores-Mir C., Nebbe B., Raboud D.W., Heo G., Major P.W. (2006). Validity, reliability, and reproducibility of plaster vs digital study models: Comparison of peer assessment rating and Bolton analysis and their constituent measurements. Am. J. Orthod. Dentofac. Orthop..

[10] Tomassetti J.J., Taloumis L.J., Denny J.M., Fischer J.R. (2001). A comparison of 3 computerized Bolton tooth-size analyses with a commonly used method. Angle Orthod..

[11] Mullen S.R., Martin C.A., Ngan P., Gladwin M. (2007). Accuracy of space analysis with emodels and plaster models. Am. J. Orthod. Dentofac. Orthop..

[12] Jeong I.D., Lee J.J., Jeon J.H., Kim J.H., Kim H.Y., Kim W.C. (2016). Accuracy of complete-arch model using an intraoral video scanner: An in vitro study. J. Prosthet. Dent..

[13] Malik J., Rodriguez J., Weisbloom M., Petridis H. (2018). Comparison of Accuracy between a Conventional and Two Digital Intraoral Impression Techniques. Int. J. Prosthodont..

[14] Kihara H., Hatakeyama W., Komine F., Takafuji K., Takahashi T., Yokota J., Oriso K., Kondo H. (2019). Accuracy and practicality of intraoral scanner in dentistry: A literature review. J. Prosthodont. Res..

[15] An X., Yang H.W., Choi B.H. (2019). Digital Workflow for Computer-Guided Implant Surgery in Edentulous Patients with an Intraoral Scanner and Old Complete Denture. J. Prosthodont..

[16] Resnick C.M., Doyle M., Calabrese C.E., Sanchez K., Padwa B.L. (2019). Is It Cost Effective to Add an Intraoral Scanner to an Oral and Maxillofacial Surgery Practice?. J. Oral Maxillofac. Surg..

[17] Grunheid T., McCarthy S.D., Larson B.E. (2014). Clinical use of a direct chairside oral scanner: An assessment of accuracy, time, and patient acceptance. Am. J. Orthod. Dentofac. Orthop..

[18] Goracci C., Franchi L., Vichi A., Ferrari M. (2016). Accuracy, reliability, and efficiency of intraoral scanners for full-arch impressions: A systematic review of the clinical evidence. Eur. J. Orthod..

[19] Camardella L.T., Breuning H., de Vasconcellos Vilella O. (2017). Accuracy and reproducibility of measurements on plaster models and digital models created using an intraoral scanner. J. Orofac. Orthop..

[20] Sfondrini M.F., Gandini P., Malfatto M., Di Corato F., Trovati F., Scribante A. (2018). Computerized Casts for Orthodontic Purpose Using Powder-Free Intraoral Scanners: Accuracy, Execution Time, and Patient Feedback. Biomed. Res. Int..

[21] Tomita Y., Uechi J., Konno M., Sasamoto S., Iijima M., Mizoguchi I. (2018). Accuracy of digital models generated by conventional impression/plaster-model methods and intraoral scanning. Dent. Mater. J..

[22] Ender A., Attin T., Mehl A. (2016). In vivo precision of conventional and digital methods of obtaining complete-arch dental impressions. J. Prosthet. Dent..

[23] Zhang F., Suh K.J., Lee K.M. (2016). Validity of Intraoral Scans Compared with Plaster Models: An In-Vivo Comparison of Dental Measurements and 3D Surface Analysis. PLoS ONE.

[24] Nedelcu R., Olsson P., Nystrom I., Ryden J., Thor A. (2018). Accuracy and precision of 3 intraoral scanners and accuracy of conventional impressions: A novel in vivo analysis method. J. Dent..

[25] Sun L., Lee J.S., Choo H.H., Hwang H.S., Lee K.M. (2018). Reproducibility of an intraoral scanner: A comparison between in-vivo and ex-vivo scans. Am. J. Orthod. Dentofac. Orthop..

[26] Jimenez-Gayosso S.I., Lara-Carrillo E., Lopez-Gonzalez S., Medina-Solis C.E., Scougall-Vilchis R.J., Hernandez-Martinez C.T., Colome-Ruiz G.E., Escoffie-Ramirez M. (2018). Difference between manual and digital measurements of dental arches of orthodontic patients. Medicine.

[27] Burhardt L., Livas C., Kerdijk W., van der Meer W.J., Ren Y. (2016). Treatment comfort, time perception, and preference for conventional and digital impression techniques: A comparative study in young patients. Am. J. Orthod. Dentofac. Orthop..

[28] Mangano A., Beretta M., Luongo G., Mangano C., Mangano F. (2018). Conventional Vs Digital Impressions: Acceptability, Treatment Comfort and Stress Among Young Orthodontic Patients. Open Dent. J..

[29] Ting-Shu S., Jian S. (2015). Intraoral Digital Impression Technique: A Review. J. Prosthodont..

[30] Kim E.-J., Hwang H.-S. (1998). Reproducibility and accuracy of tooth size measurements obtained by the use of computer. Korean J. Orthod..

[31] Flugge T.V., Schlager S., Nelson K., Nahles S., Metzger M.C. (2013). Precision of intraoral digital dental impressions with iTero and extraoral digitization with the iTero and a model scanner. Am. J. Orthod. Dentofac. Orthop..

[32] Gul Amuk N., Karsli E., Kurt G. (2019). Comparison of dental measurements between conventional plaster models, digital models obtained by impression scanning and plaster model scanning. Int. Orthod..

[33] Muallah J., Wesemann C., Nowak R., Robben J., Mah J., Pospiech P., Bumann A. (2017). Accuracy of full-arch scans using intraoral and extraoral scanners: An in vitro study using a new method of evaluation. Int. J. Comput. Dent..

[34] Muller P., Ender A., Joda T., Katsoulis J. (2016). Impact of digital intraoral scan strategies on the impression accuracy using the TRIOS Pod scanner. Quintessence Int..

[35] Shahen S., Carrino G., Carrino R., Abdelsalam R., Flores-Mir C., Perillo L. (2018). Palatal volume and area assessment on digital casts generated from cone-beam computed tomography scans. Angle Orthod..

