# Supplementary material for: Evaluation of the Reliability, Reproducibility and Validity of Digital Orthodontic Measurements Based on Various Digital Models among Young Patients
Source: J Clin Med. 2020 Aug 24;9(9):2728. doi: 10.3390/jcm9092728 (PMC7564383; doi:10.3390/jcm9092728)
Supplement: Supplementary file 1 [file jcm-09-02728-s001.pdf]

Journal of
Clinical Medicine

MDPI

Supplementary material

Evaluation of the Reliability, Reproducibility and Validity of Digital Orthodontic Measurements Based on Various Digital Models among Young Patients

Seo-Hyun Park [1,2,†], Soo-Hwan Byun [2,3,4,†], So-Hee Oh [1,2,3], Hye-Lim Lee [1], Ju-Won Kim [2,3,4], Byoung-Eun Yang [2,3,4,*] and In-Young Park [2,3,5,*]

1 Division of Pediatric Dentistry, Hallym University Sacred Heart Hospital, Anyang 14066, Korea; park070676@gmail.com (S.-H.P.); colfman@hanmail.net (S.-H.O.); onlylove0210@naver.com (H.-L.L.)
2 Graduate School of Clinical Dentistry, Hallym University, Chuncheon 24252, Korea; purheit@hallym.or.kr (S.-H.B.); kjw9199@hallym.or.kr (J.-W.K.)
3 Institute of Clinical Dentistry, Hallym University, Chuncheon 24252, Korea
4 Division of Oral and Maxillofacial Surgery, Hallym University Sacred Heart Hospital, Anyang 14066, Korea
5 Division of Orthodontics, Hallym University Sacred Heart Hospital, Anyang 14066, Korea
* Correspondence: face@hallym.ac.kr (B.-E.Y.); park.iy2875@gmail.com (I.-Y.P.); Tel.: +82-31-380-3870 (B.-E.Y. & I.-Y.P.)
† These authors contributed equally to this work.

Table 1. Reliability of tooth widths and arch lengths in each group.

Verification	P group		MSD group		ISD group	
	coefficients	*p* value	coefficients	*p* value	coefficients	*p* value
Tooth width (#15 - #45)	0.989 – 0.996	<0.0001[a]	0.996 – 0.999	<0.0001[a]	0.992 – 0.998	<0.0001[a]
Maxilla						
AAL	0.998	<0.0001[a]	0.996	<0.0001[a]	0.996	<0.0001[a]
LAL	0.998		0.997		0.994	
RAL	0.998		0.991		0.994	
CAL	0.998		0.999		0.995	
Mandible						
AAL	0.989	<0.0001[a]	1.000	<0.0001[a]	0.982	<0.0001[a]
LAL	0.995		0.998		0.984	
RAL	0.993		0.999		0.984	
CAL	0.998		0.999		0.995	

Pearson's correlation coefficient (a: p<0.0001). FDI 2-digit notation system is used to identify teeth. P = Plaster model, MSD = model scanned digital model, ISD = intraoral scanned digital model, AAL = Anterior arch length, LAL = Left arch length, RAL = right arch length, CAL = Curved arch length.

Table 2. Reproducibility of the tooth widths and arch lengths in each group.

Verification	P group	MSD group	ISD group	Verification	P group	MSD group	ISD group
#15	0.873[b]	0.953[a]	0.945[a]	#35	0.796[b]	0.946[a]	0.938[a]
#14	0.839[b]	0.936[a]	0.920[a]	#34	0.893[b]	0.957[a]	0.935[a]
#13	0.915[a]	0.960[a]	0.910[a]	#33	0.825[b]	0.940[a]	0.915[a]
#12	0.869[b]	0.948[a]	0.925[a]	#32	0.866[b]	0.963[a]	0.926[a]

#11	0.871[b]	0.965[a]	0.848[b]	#31	0.763[b]	0.893[b]	0.842[b]
#21	0.834[b]	0.967[a]	0.945[a]	#41	0.817[b]	0.911[a]	0.845[b]
#22	0.880[b]	0.932[a]	0.883[b]	#42	0.811[b]	0.936[a]	0.907[a]
#23	0.865[b]	0.972[a]	0.957[a]	#43	0.879[b]	0.937[a]	0.915[a]
#24	0.860[b]	0.946[a]	0.922[a]	#44	0.857[b]	0.961[a]	0.944[a]
#25	0.842[b]	0.950[a]	0.934[a]	#45	0.854[b]	0.955[a]	0.934[a]
Maxilla				Mandible			
AAL	0.914[a]	0.832[b]	0.628[c]	AAL	0.880[b]	0.818[b]	0.774[b]
LAL	0.936[a]	0.725[c]	0.724[c]	LAL	0.777[b]	0.807[b]	0.583[c]
RAL	0.956[a]	0.827[b]	0.753[b]	RAL	0.851[b]	0.792[b]	0.554[c]
CAL	-	0.937[a]	0.925[a]	CAL	-	0.937[a]	0.925[a]

Intraclass correlation coefficient (a: ICC≥0.9, b: 0.9>ICC≥0.75, c: 0.75>ICC≥0.5). P = Plaster, MSD = model scanned digital model, ISD = intraoral scanned digital model, AAL = Anterior arch length, LAL = Left arch length, RAL = right arch length, CAL = Curved arch length.
